# Geographical origin of chronic Chagas disease patients in Brazil impacts the performance of commercial tests for anti-*T. cruzi* IgG

**DOI:** 10.1590/0074-02760210032

**Published:** 2021-05-26

**Authors:** Amadeo Sáez-Alquezar, Angela Cristina Verissimo Junqueira, Andressa da Matta Durans, André Valpassos Guimarães, José Abol Corrêa, José Borges-Pereira, Patrícia Lago Zauza, Pedro Hernan Cabello, Pedro Albajar-Viñas, David William Provance, José Rodrigues Coura

**Affiliations:** 1 Sociedade Brasileira de Análises Clínicas, Programa Nacional de Controle de Qualidade, Rio de Janeiro, RJ, Brasil; 2 Fundação Oswaldo Cruz-Fiocruz, Instituto Oswaldo Cruz, Laboratório de Doenças Parasitarias, Rio de Janeiro, RJ, Brasil; 3 Fundação Oswaldo Cruz-Fiocruz, Centro de Desenvolvimento Tecnológico em Saúde, Rio de Janeiro, RJ, Brasil; 4 Fundação Oswaldo Cruz-Fiocruz, Instituto Oswaldo Cruz, Laboratório Interdisciplinar de Pesquisas Médicas, Rio de Janeiro, RJ, Brasil; 5 Fundação Oswaldo Cruz-Fiocruz, Instituto Oswaldo Cruz, Laboratório de Genética Humana, Rio de Janeiro, RJ, Brasil; 6 Universidade do Grande Rio, Laboratório de Genética, Rio de Janeiro, RJ, Brasil; 7 World Health Organization, Department of Control of Neglected Tropical Diseases, Geneva, Switzerland

**Keywords:** Trypanosoma cruzi, human Chagas disease, serological diagnostic test, immunoassays, International Biological Reference Standards

## Abstract

**BACKGROUND:**

Chagas disease, caused by *Trypanosoma cruzi*, affects nearly six million people worldwide. Various serological tests have been developed for its diagnosis.

**OBJECTIVE:**

Examine the performance of a set of commercial immunological assays in relation to the geographical origin of the patient sample comparing four states of Brazil: Amazonas (AM), Mato Grosso do Sul (MS), Minas Gerais (MG) and Piauí (PI).

**METHODS:**

Seven immunoassays were employed to detect anti-*T. cruzi* IgG antibodies in 379 patient samples that had been previously diagnosed using the two-step protocol required by the Brazilian Ministry of Health.

**FINDINGS:**

A significant variation in the percent reactive was calculated for the samples from AM and MS, while the PI and MG showed a significant variation in the percent non-reactive. The average reactivity index was significantly higher for samples from the states of PI and MG states than AM and MS.

**MAIN CONCLUSIONS:**

All tests presented a satisfactory performance overall. Yet, variations were observed that were associated to the region of origin of the samples. Our analyses suggest that future evaluations of immunoassays should include a sampling of sera from regions where the test will be applied in addition to the available International Biological Reference Standards.

Chagas disease is primarily a tropical disease caused by infections of the parasite *Trypanosoma cruzi*. It is considered endemic in 21 countries of Latin America and current estimates suggest around six million people are infected with nearly 14,000 deaths per year being attributed to their infection.^1^ The classic mode of transmission is through the deposit of parasites from infected triatomines after a blood meal.^2^ This vector is characteristic to endemic regions, yet there are other forms of transmission that include blood transfusion, organ transplants, congenital and oral uptake through the ingestion of freshly prepared juices and foods contaminated with infected insects.^1^ ^,^ ^3^ Transmission through laboratory accidents is also possible although limited to a restricted set of professionals.

Extensive efforts have been made to reduce infections rates in endemic regions through control measures for the insect vectors.^1^ ^,^ ^4^ ^,^ ^5^ A major success of these programs was interrupted of *Triatoma infestans*, the primary insect vector in Brazil, Uruguay and Chile.^6^ ^,^ ^7^ ^,^ ^8^ In the other endemic countries, the interruption of *T. infestans* is still ongoing. However, over the last few decades, there has been a contingent of migrants from endemic areas to non-endemic areas, including other continents, which represent the emergence of new risks for some sectors of the countries and regions, that have received them such as blood transfusion and organ transplantation as well as the occurrence of congenital Chagas. The countless efforts made in recent decades by governmental institutions and international entities have contributed significantly to a reduction in the rates of vector transmission and blood transfusion. More recent actions have been directed towards an interruption of vertical transmission from infected mothers to newborns and for the treatment of infected people, both in children and in adults in the chronic phase of the disease.

To control all these actions, the use of serological tests is essential to correctly identify people infected with *T. cruzi* and also specific cases, such as in the diagnosis of newborns or in the evaluation of therapeutic efficacy or the efficiency of molecular tests. Data in the literature suggest that less than 10% of persons infected in endemic areas are diagnosed.^9^ ^,^ ^10^ ^,^ ^11^ It is considered that the expansion of testing in populations at risk, such as women of childbearing age in endemic areas, may benefit subsequent prevention and treatment actions.

There is a wide array of commercial serological tests available on the international market that employ both old and new methodologies. Each approach has its advantages and disadvantages that can direct their choice for the appropriate use in different situations according to access to available resources.^12^ ^,^ ^13^ ^,^ ^14^ ^,^ ^15^ ^,^ ^16^ Within the so-called conventional tests, there are assays for indirect haemagglutination (HAI), indirect immunofluorescence (IFI) and enzyme-linked immunosorbent assay (ELISA) tests that use parasitic lysate (lys) as antigenic fractions. The newer methodologies are based on the ELISA format, but use as antigenic fractions, recombinant proteins (rec) and/or synthetic peptides (ps). In addition, some use Chemiluminescence and electro Chemiluminescence tests (CMIA/eCLIA) in combination with similar types of antigenic fractions of rec and ps. Lastly, there are rapid diagnostic tests that can be extremely useful under increasingly frequent circumstances due to their speed and improvements in the quality of the results, which have improved in specificity and sensitivity over time.

Other diagnostic possibilities are the use of direct (TPD) and indirect (TPI) parasitological tests, each of which depends on the presence of circulating parasites in the biological sample to be analysed. TPDs are indispensable in cases of acute Chagas as there are no distinction between anti-*T. cruzi* specific antibodies generated in the acute phase and later during the chronic phase.^17^ However, this type of analysis is highly dependent on the training and experience of the technician responsible for evaluating the blood smear fields. Rather than directly observing parasites, polymerase chain reaction (PCR) techniques can be used to amplify segments of the parasite genome. While it depends on the presence of circulating parasites, the volume of blood that can be prepared for analysis is much greater than that observed in a blood smear. Combined with the sensitivity of PCR reactions, this approach has been useful in certain circumstances. However, it still does not have adequate standardisation, requires more sophisticated equipment and trained personnel.

Overall, serological tests have the greatest potential to be applied at a scale necessary to combat the impact of *T. cruzi* infections on the global society. Yet, to date, no single test has met the performance profile required to be considered a “gold standard. In a previous analysis of commercial test performance,^13^ the International Biological Standards of the WHO were applied to show a difference based on the two geographical regions that encompassed by the sera included in the two standards. In Brazil, due to its continental dimensions, we postulated that there could be differences in the behavior of serological tests in relation to different regions of the country. The variances could reflect several factors such as the genetic diversity of the infecting agent and the clinical conditions of infected individuals. Here, we present our analysis on the performance of eight serological tests on serum samples from individuals with previous serological diagnosis of Chagas infection, or not, that live in four states of Brazil, Amazonas, Piauí, Minas Gerais and Mato Grosso do Sul where Chagas disease is endemic.

SUBJECTS and METHODS

*Ethical considerations for the use of human serum* - All patient serum samples used in the present study were previously obtained from individuals who participated in sectional and longitudinal epidemiological studies carried out in the referred areas at intervals of 9 to 13 years.^18^ ^,^ ^19^ ^,^ ^20^ All sample collections and experiment use were pre-approved by the ethical committee of the Oswaldo Cruz Institute of Fiocruz (CEP 289/05 and Protocol number 0019.0.009.000-07).

*Patient Samples* - Four states of Brazil were chosen to represent the Northern (Amazonas - AM), Northeastern (Piauí - PI), Southeastern (Minas Gerais - MG) and Central Western (Mato Grosso do Sul - MS) geographical regions of Brazil (Fig. 1). Serological samples were collected in the municipalities of Barcelos in AM (n = 79), João Costa in PI (n = 100) and Virgem da Lapa in MG (n = 100). In MS, collections were performed in the Rio Verde Sanitary District that encompasses Alcinópolis (n = 11), Bandeirantes (n = 09), Camapuã (n = 20), Corguinho (n = 05), Coxim (n = 18), Pedro Gomes (n = 07), Rio Negro (n = 06), Rio Verde (n = 11), Rochedo (n = 02), São Gabriel (n = 08), Sonora (n = 02) and Jaraguari (n = 01). The average ages of participants from the four states of AM, PI, MG and MS were 33, 58, 56 and 55 years, respectively. The percent ratio of women to men was 43:57 in AM, 56:44 in PI and 63:37 in both MG and MS.

**Fig. 1:**
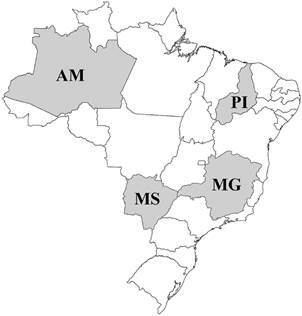
map of Brazil for patient serum collection sites with the states shaded (AM - Amazonas; PI - Piauí; MG - Minas Gerais; MS - Mato Grosso do Sul).

*Diagnostic assays for the detection of T. cruzi specific antibodies* - At the time of collection, all serum samples were assayed in the Laboratory of Parasitic Diseases (LPD) of the Oswaldo Cruz Institute (IOC) in the Oswaldo Cruz-RJ Foundation (Fiocruz) using two independent protocols, as established by the Brazilian Ministry of Health,^21^ which employed Chagas IFI kits (Biomanguinhos, RJ, Brazil) comprised of epimastigote forms of *T. cruzi* cultivated in liver infusion tryptone and ELISA Chagas 3.0 (Wiener Laboratory, Argentina) containing recombinant antigens. Both tests were utilised before their expiration dates. Samples were stored at -20ºC until the start of the current study. A subset of 100 of the of the original serum panel from PI, MG and MS were randomly chosen while all 79 samples from AM were employed. Sera was processed in the laboratory of the National Quality Control Program (PNCQ-RJ, Brazil) and assayed by the eight serological tests (Table I). Six kits were based on the ELISA methodology (Gold; Bioschile; Biokit; D-Med; BioMérieux; Wiener 4.0), one used chemiluminescence with magnetic spheres (CMIA; Abbot Architect) and the other used a western blot format (TESA Blot, BioMérieux). All tests were fastidiously performed according to the manufacturer’s instructions and before their expiration date. Today the BioMérieux ELISA kit has been discontinued. For the ELISA assays, a Columbus Microplate Washer automatic plate washer (TECAN, Männedorf, CH) was used and a Sunrise™ ELISA reader (TECAN) was used for the measurement of optical densities. The chemiluminescent magnetic immunoassays were conducted on an automated Architect i2000 platform (Abbott, Illinois, USA).

**TABLE I:** List of the commercial tests utilised, their format and the components used to capture *Trypanosoma cruzi* antibodies

Commercial test	Method	Antigenic target	Batch	Reader/Analyser
Gold	ELISA	Lys + Rec	CHA084A	TECAN
BiosChile	ELISA	Lys	1H110388	TECAN
D-MED	ELISA	Lys	110102	TECAN
BioMérieux	ELISA	Lys	1203106006	TECAN
Biokit	ELISA	Rec	L-1411	TECAN
Wiener	ELISA	Rec	1109075160	TECAN
Abbott/Architect	CMIA	Rec	14857LI00	Architect i2000
TESA Blot (BioMérieux)	WB	Ag Trypo	1204106150	N/A

ELISA: enzyme linked immunosorbent assay; CMIA: chemiluminescence magnet immunoassay; WB: western blot; Lys: total *T. cruzi* lysate; Rec: recombinant *T. cruzi* proteins; Ag Trypo: antigens excreted or secreted by trypomastigote forms of *T. cruzi*. N/A: not applicable.

*Statistical analysis* - The differences in the proportions of the qualitative results of the samples were submitted to the Mantel-Haenszel chi-square test and the differences in the means of the reactivity index (OD/CO) of the tests were subjected by Analysis of variance (ANOVA) Two-Way,^22^ which considered the individual effects on OD/CO between the commercial assays, the geographical region of collection (states of Brazil) and the interaction between these two variables. In both statistical analyses, a 95% confidence level was considered for a p ≤ 0.05.

RESULTS

A previous longitudinal, sectional epidemiology study provided a unique opportunity to evaluate the performance of eight commercially available tests for the detection of anti-*T. cruzi* antibodies in the serum of patients suspected with chronic Chagas disease in relation to four different geographical regions (states) of Brazil. To represent the areas covered by the states of PI, MG and MS areas, 100 samples were chosen by random numbers that covered the identifiers of the serum records in the initial diagnostic analysis of the sera that was performed in the Laboratory of Parasitic Diseases of IOC/Fiocruz-RJ. A similar approach was used to represent the AM area with the number of samples restricted to 79. A total of 379 patient samples were analysed.

The selection included reactive (seropositive by both two assays) and non-reactive (seronegative by both assays) for a *T. cruzi* infection as well as indeterminate that presented reactivity by one test and non-reactive by the other. Table II shows the distribution by the state of Brazil for the 234 reactive, 109 non-reactive and the 36 indeterminant samples. From this initial analysis, there appeared to be differences between the percentages of seropositive samples, seronegative samples and serum-divergent samples according to the area of origin. The highest percentage of indeterminate results was found for samples collected from patients in the state of Amazonas and the lowest was for collections in Piauí.

**TABLE II:** Initial diagnosis of patient samples using a two, independent assay protocol

	AM* n (%)	PI* n (%)	MG* n (%)	MS* n (%)	Total n (%)		
IFI (+) & ELISA (+)	41 (52%)	86 (86%)		64 (64%)		43 (43%)	234 (61.7%)
IFI (-) & ELISA (-)	15 (18.9%)	14 (14%)		28 (28%)		52 (52%)	109 (28.7%)
IFI (+) & ELISA (-)	17 (21.5%)	0		8 (8%)		5 (5%)	30 (7.9%)
IFI (-) & ELISA (+)	6 (7.6%)	0		0		0	6 (1.7%)
Total	79	100		100		100	379

*: site of collection by state of Brazil (AM - Amazonas; PI - Piauí; MG - Minas Gerais; MS - Mato Grosso do Sul); IFI: indirect immunofluorescence; ELISA: enzyme linked immunosorbent assay; (+) reactive; (-) non-reactive.

When the panel of sera were tested in the current study by the seven commercially available kits, 221 continued to present reactivity in a majority of the tests (Table III). This translates to 94.4% of the 234 samples that were diagnosed to be reactive in the initial diagnosis. Of the 109 non-reactive samples, 104 (95.4%) were also non-reactive in the serological profile tests across the commercial kits. To corroborate these results with a different format, the TESA blot was used as a complementary test. From the number of samples determined to be reactive, 95.9% were validated to maintain their reactive over the time of storage. The range of percentages from 88.9% in AM to 97.6% in PI and MS were determined to be a statistically non-significant difference (X^2^ = 1.767; p = 0.183). For the 36 indeterminant samples, 16 (44.4%) were negative in the majority of commercial tests and 20 (55.6%) showed reactivity (data not shown). Overall, it appeared as though the antibody titer in the patient samples were not diminished based on the comparison of the initial tests for reactivity to the serological kits treated as a group.

**TABLE III:** Percent of immunoassay reactive samples in the majority of commercial tests and their detection by the TESA blot

State of sample origin	Number of samples	Number (percent) reactive	Number (percent) reactive by TESA
Amazonas	79	27 (65.8%)	24 (88.9%)
Piauí	100	82 (95.3%)	80 (97.6%)
Minas Gerais	100	70 (91.4%)	67 (95.7%)
Mato Grosso do Sul	100	42 (97.6%)	41 (97.6%)
Totals	379	221 (94.4%)	212 (95.9%)

By considering the percentage of samples that displayed a positive reactivity for anti-*T. cruzi* IgG from each commercial test in the 234 biological samples to the initial diagnosis (Fig. 2), it can be seen that the values fluctuated from 83.8% to 94.0% between the kits (Table IV). The differences in reactive were statistically significant (X^2^ = 17.521; p = 0.000030), which indicated a different level of sensitivity for the different tests. Taking as a reference the results of the serology in the initial phase, an analysis of the percentages of reactive (sensitivity) of the tests of the serological profile was performed according to the area of origin of the samples. Significant differences in the values were obtained with the samples of the AM and MS states, indicating different sensitivities of the tests in these areas. In contrast, the percentages obtained in the states of PI and MG showed no significant difference, with an emphasis on the values of 100% for all tests in MG, indicating an equal performance in the sensitivity of the tests applied in this area.

**Fig. 2:**
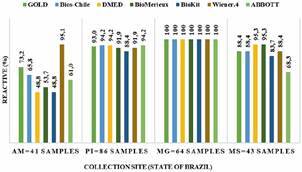
percentage of commercial tests that were reactive for anti-*Trypanosoma cruzi* IgG in sera reactive in the initial diagnosis according to the Brazilian state were collections were conducted. The panel of sera determined to be positive for anti-*T. cruzi* IgG by the two-step protocol and their reactive (%) in each of the commercial kits for anti-*T. cruzi* IgG to the state of origin of the biological samples. MG - Minas Gerais; PI - Piauí; MS - Mato Grosso do Sul; AM - Amazonas.

**TABLE IV:** Number of reactive and non-reactive sera according to the individual commercial tests in relation to initial diagnosis

Commercial test	Number reactive (% of initial diagnosis*)	Number non-reactive (% of initial diagnosis)
GOLD	212 (90.6)	104 (95.4)
Bios-Chile	210 (89.7)	106 (97.3)
DMED	206 (88.0)	102 (93.6)
BioMerieux	206 (88.0)	104 (95.4)
BioKit	196 (83.8)	101 (92.7)
Wiener.4	220 (94.0)	104 (95.4)
ABBOTT	198 (84.6)	86 (78.9)
Statistical analysis	X^2^ = 17.521; p = 0.00003	X^2^ = 3.193; p = 0.073

*: from the initial diagnosis, 234 sera were positive and 109 were negative.

For specificity, the percentage of non-reactive results for the 109 samples was determined in relation to the number of non-reactive samples from the initial analysis (Fig. 3). The analysis of the percentage of non-reactive (specificity) of the tests reveals 100% for all tests in the samples of AM; significant difference in the values of PI and MG, and in MS only the Abbott test proved to be significantly lower in comparison with the other tests (Fig. 3). A statistically non-significant difference (X^2^ = 3.193; p = 0.073) was obtained, despite the lower value of the Abbott Laboratory’s CMIA test, suggesting that the array of commercial tests displayed a similar level of specificity.

To further evaluate the performance of each serological test in relation to the geographical origin of the biological samples, the mean values of their reactive indices were determined by the ratio of the optical density to cutoff and scatter plotted according to the group of states (Fig. 4). Five of the commercial tests displayed higher values in samples obtained from PI and MG that were significantly lower in samples from AM and MS that indicated a regional difference in their performance to measure anti-*T. cruzi* IgG levels. Two of the commercial tests displayed lower reactivity index in PI and MS that were in the range of the other five kits in AM and MS.

**Fig. 3:**
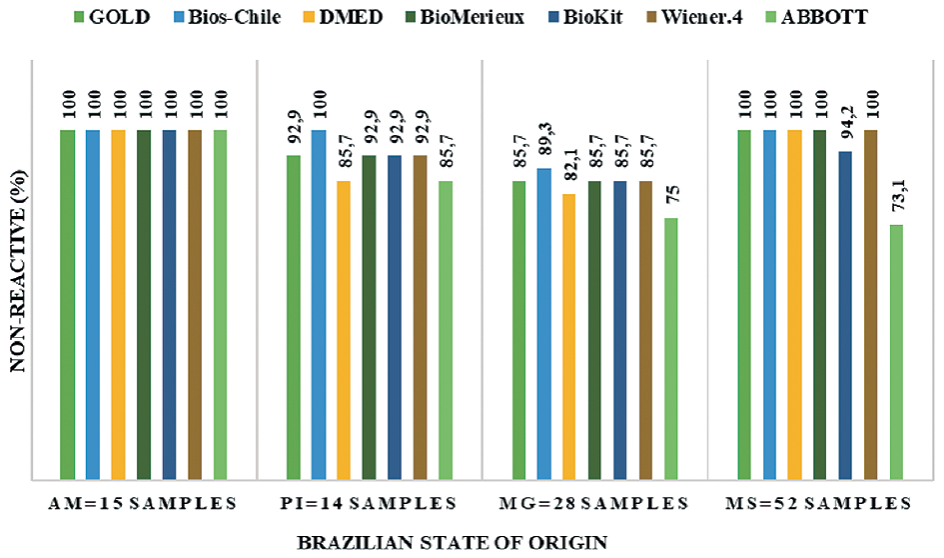
percentage of results from each commercial test that was non-reactive for anti-*T. cruzi* IgG of the initial diagnosis according to the Brazilian state of origin. The panel of sera determined to be negative for anti-*T. cruzi* IgG by the two-step protocol and their non-reactive (%) in each of the commercial kits for anti-*T. cruzi* IgG to the state of origin of the biological samples. MG - Minas Gerais; PI - Piauí; MS - Mato Grosso do Sul; AM - Amazonas.

**Fig. 4:**
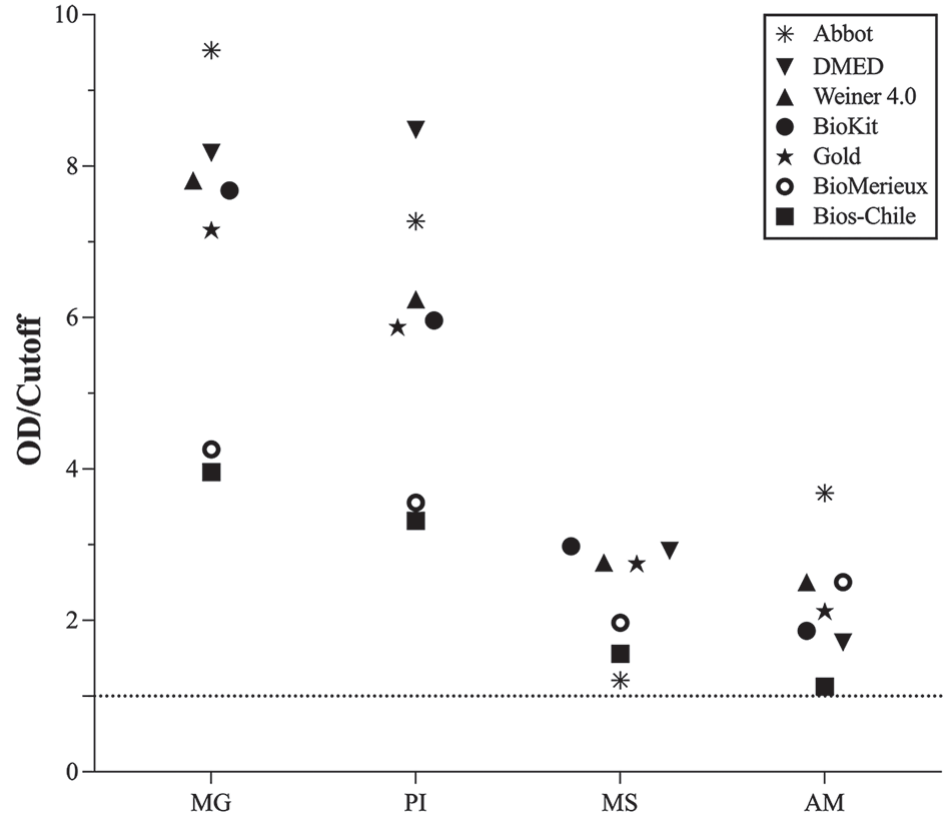
the reactivity index (OD/CO) measured from each test in the detection of anti-*T. cruzi* IgG according to the Brazilian state of origin. The mean optical density measured by each kit for reactivity biological samples were divided by the cutoff to generate the RI and plotted according to the state of Brazil where the sample was collected. MG - Minas Gerais; PI - Piauí; MS - Mato Grosso do Sul; AM - Amazonas.

Discussion

The previous realisation of sectional and longitudinal clinical-epidemiological studies on chronic Chagas disease in the states of AM, PI, MG and MS provided an excellent foundation for the current study. It generated a panel of sera that had been collected under similar conditions by a single group of researchers. It also included access to clinical information and a diagnosis of each sera as determined through two independent assays, indirect immunofluorescence and ELISA. The conclusions of these studies showed significant regional differences in the morbidity of Chagas disease with a higher prevalence and severity of cardiac and digestive forms among patients from PI and MG that was notably lower among patients from AM and MS.^16^ ^,^ ^18^ ^,^ ^19^ ^,^ ^20^ ^,^ ^23^ ^,^ ^24^ ^,^ ^25^

The choice of the seven immunoassays utilised in the present study was based on their availability on the market and their approval by ANVISA, the responsible Brazilian regulatory agency. In addition, these same kits had been used in a previous evaluation using the two WHO international biological reference standards for *T. cruzi* antibodies that showed a performance difference between the origins of the sera used to generate the standards.^26^ The whole of Brazil was represented in the reference standard for which all of the commercial tests showed a lower sensitivity. As Brazil is a geographically expansive country and endemic for different strains of *T. cruzi*, we reasoned that the geographical differences observed with the WHO standards could extend to subregions of the country, represented as the states where patient samples were collected.

The initial concern was that the storage of the biological samples would lead to a loss of antibody titer. The data presented in Table II suggested that this was not the case as the percent of samples that remained reactive in a majority of the commercial tests was 94.4%. More importantly, the percent reactivity by the TESA blot was 95.9%, which is notable due to the normally lower sensitivity of this assay compared to ELISAs, the format of six of the seven commercial kits. The other format was a chemiluminescence assay (CMIA).

The TESA test is considered a complementary test for specificity that consists of the fractionation and separation of antigens excreted and secreted from trypomastigote cultures whose results are interpreted based on a visual evaluation.^18^ ^,^ ^19^ ^,^ ^20^ Here, the specificity of the commercial tests was considered good based on the analysis of the non-reactive samples. All of the samples from AM and MS that were initially considered non-reactive remained non-reactive and only one from PI showed weak reactivity by the commercial tests. For the 28 samples from MG, five that were non-reactive by the initial tests were reactive in the commercial tests. The percent of samples that agree with the initial non-reactive diagnosis from the individual tests in relation to the region of origin of the biological samples showed good consistency with the exception of the CMIA that should the lowest percentage.

The practical use of the commercial tests involves the use of a binary output, reactive or non-reactive. It is from the use of two or more tests that a result can be considered divergent, reactive in one test and non-reactive in another. Here, the number of samples with divergent results for reactivity between the initial diagnosis and the commercial kits varied by the geographical site of collection. All 64 samples from MG that were reactive by the initial tests also showed reactivity in the commercial kits while only two of the 43 initially reactive samples from MS showed non-reactive. In the PI samples, of the 86 that were reactive for the two initial tests, 82 maintained reactive in the commercial kits. The AM samples showed the greatest divergence. Of the 41 reactive by the initial two tests, only 26 remained reactive.

The divergence in the AM and MS samples was more apparent when displaying the individual positivity scores for each commercial test in Fig. 2. The principal range of concurrence to the initial diagnosis of the AM samples was from 48.8% to 73.2 with one outlier at 95.1% (Wiener 4.0). For the MS samples, positivity ranged from a low of 68.3% to 95.3%. These results suggested a difference in the sensitivity to detect anti-*T. cruzi* IgG antibodies that was related to the geographical region of Brazil where the samples were collected. This conclusion was reinforced by examining the level of reactivity by plotting the mean ratio of optical density to cutoff value for each commercial test to the state of origin of the samples in Fig. 4. Five of the commercial tests displayed levels several fold higher than the ratio of 1 for samples collected in MG and PI, whereas the median levels were much lower, although reactive, for MS and AM. The other two commercial tests showed the same trend, but not to the same extent.

There are a number of possible explanations for the apparent lower sensitivity of the commercial tests for the states of MS and AM. These include collection conditions such as ambient temperature and humidity, transport errors, processing errors, storage errors that reduced antibody titer, lack of internal quality controls and/or variations in the performance of the kit lots used. The differences in sensitivity could reflect the performance of the components used to capture anti-*T. cruzi* antibodies in the different commercial kits. Three of the ELISA formats employ antigens composed of the total lysate prepared from parasites grown in LIT, which would be the epimastigote form of the parasite. Three others incorporate antigens consisting of recombinant proteins and one uses a combination of lysate with recombinant proteins. Another possibility is differences in the immune response of the patient related to the genetics of the demographics, the strain of *T. cruzi* responsible for the infection or a combination of the two.

It is interesting to note that the behavior of the immunoassays, with respect to reactivity or non-reactivity, was homogeneous in practically all samples analysed, with no discrepancies between them. However, the major impetus for performing these evaluations persists: the absence of a gold standard immunological assay for the diagnosis of a *T. cruzi* infection. This is reflected by the number of indeterminate diagnoses from the initial testing. Of the 30 samples that were initially reactive only in the IFI test, 25 were non-reactive and five were reactive by the commercial tests. The six samples that had a non-reactive result in the initial IFI test and a reactive result in the ELISA test, all presented with non-reactive results by the commercial tests. It should be noted that the IFI test, due to its characteristics of using reagents from various sources and visual reading, has been considered a source of false positive results in several screening studies.^15^ ^,^ ^16^ Other false positives can occur from cross-reactivity, in particular with *Leishmania*.^27^ While the samples analysed here were not tested for antibodies against *Leishmania*, the consistency between assay results that use different targets suggests little cross reactivity.

Ultimately, the difference in sensitivity detected among the commercial tests related to the geographical origin of the biological sample can be considered a measure of the diverse regional robustness of the test. All of the tests showed a limited robustness for the samples from AM and MS that could be undermined further by any or all of the extenuating circumstances surrounding the collection of a biological sample. Developers and regulators should be encouraged to evaluate the performance of new kits that go beyond the pathogen to include sera panels that encompass possible regional influences on performance.
